# Additive Manufacturing of Nerve Decellularized Extracellular Matrix-Contained Polyurethane Conduits for Peripheral Nerve Regeneration

**DOI:** 10.3390/polym11101612

**Published:** 2019-10-04

**Authors:** Yi-Wen Chen, Chien-Chang Chen, Hooi Yee Ng, Ching-Wen Lou, Yueh-Sheng Chen, Ming-You Shie

**Affiliations:** 1 Graduate Institute of Biomedical Sciences, China Medical University, Taichung 40447, Taiwan; evinchen@gmail.com; 2 3D Printing Medical Research Institute, Asia University, Taichung 40447, Taiwan; 3 3D Printing Medical Research Center, China Medical University Hospital, Taichung 40447, Taiwan; m0975371198@gmail.com (C.-C.C.); hooiyeen@gmail.com (H.Y.N.); 4 School of Medicine, China Medical University, Taichung 40447, Taiwan; 5 Department of Bioinformatics and Medical Engineering, Asia University, Taichung 40447, Taiwan; cwlou@asia.edu.tw (C.-W.L.); yuehsc@mail.cmu.edu.tw (Y.-S.C.); 6 Biomaterials Translational Research Center, China Medical University Hospital, Taichung 40447, Taiwan; 7 Lab of Biomaterials, School of Chinese Medicine, China Medical University, Taichung 40447, Taiwan; 8 School of Dentistry, China Medical University, Taichung 40447, Taiwan

**Keywords:** nerve regeneration, polyurethane, extracellular matrix, dopamine, digital light processing

## Abstract

The nervous system is the part of our body that plays critical roles in the coordination of actions and sensory information as well as communication between different body parts through electrical signal transmissions. Current studies have shown that patients are likely to experience a functional loss if they have to go through a nerve repair for >15 mm lesion. The ideal treatment methodology is autologous nerve transplant, but numerous problems lie in this treatment method, such as lack of harvesting sites. Therefore, researchers are attempting to fabricate alternatives for nerve regeneration, and nerve conduit is one of the potential alternatives for nerve regeneration. In this study, we fabricated polyurethane/polydopamine/extracellular matrix (PU/PDA/ECM) nerve conduits using digital light processing (DLP) technology and assessed for its physical properties, biodegradability, cytocompatibility, neural related growth factor, and proteins secretion and expression and its potential in allowing cellular adhesion and proliferation. It was reported that PU/PDA/ECM nerve conduits were more hydrophilic and allowed enhanced cellular adhesion, proliferation, expression, and secretion of neural-related proteins (collagen I and laminin) and also enhanced expression of neurogenic proteins, such as nestin and microtubule-associated protein 2 (MAP2). In addition, PU/PDA/ECM nerve conduits were reported to be non-cytotoxic, had sustained biodegradability, and had similar physical characteristics as PU conduits. Therefore, we believed that PU/PDA/ECM nerve conduits could be a potential candidate for future nerve-related research or clinical applications.

## 1. Introduction

The nervous system is the part of our body that plays critical roles in the coordination of actions and sensory information as well as communication between different body parts through electrical signal transmissions. It works by coordinating inputs from different origins and turn them into actions and autonomic outputs in response to environmental changes. Therefore, severe neurological dysfunction or damage would lead to disabilities that cause major negative impacts on the quality of a patient’s life [1]. The human nervous system can be categorized into two broad components, one being the central nervous system (CNS) and the other being the peripheral nervous system (PNS). CNS comprises the brain and the spinal cord, while the PNS encompasses the rest of the system other than the brain and spinal cord [2]. Patients that are involved in major traumas often suffer from peripheral nerve damages due to the extent of the impact, and such damages usually include nerve compression, nerve lesions, or tear, or even ischemic injuries [3]. Other reasons that can cause peripheral nerve damage include surgical procedures and other co-morbidities. These damages would subsequently lead to various kinds of neurological dysfunction and diseases. Furthermore, studies have shown that approximately 2.8% of the patients still claim to have peripheral nerve issues after receiving treatment, some suffer from neuropathic pain and permanent dysfunction, and some patients even experience permanent disability after years of recovery [4]. The rate of success of peripheral nerve treatments has improved significantly with the advancement of medical technology and knowledge. However, there remains a huge gap between such recoveries and complete recovery of neural functions due to the limited regenerative capabilities of the nerves. To date, the incorporation of regenerative medicine into peripheral nerve treatments remains a challenge due to major obstacles, including lengthy regenerative duration, limited sources of nerves for autologous transplantations, as well as the rejection of autologous transplantations [5]. Therefore, scientists are always in search for novel solutions to overcome these challenges. Since the past decade, tissue engineering has been seen as a potential substitute for nerve transplantations and it has since become an important research topic in the field of nerve regeneration [6].

Clinically, it is a major challenge to attempt to regenerate or replace severe nerve damages. Temporary loss of nerve functions is inevitable after any kind of nerve damages as the communications between the nerves have been interrupted. Previous cases showed that patients who underwent repair of >15 mm nerve defects often claimed to have functional loss even after recovery. In addition, neural lesions involving distances of >15 mm may significantly decrease the regeneration capabilities of the nerves due to the disruption of endo-neural contacts between proximal and distal ends [4]. Thus, nerve conduits are commonly used in clinical settings to overcome this issue. Some of the most widely used conduits include the autologous conduits, the non-polymer conduits (such as those that are made up of stainless steel and silicon), and biodegradable conduits (such as those that are made up of natural polymer) [7,8,9]. However, of all the options, autologous nerve transplantations are the current mainstream treatment method for nerve injuries. Nerve grafts from the autologous origin are optimal because they have been proved to not only protect the damaged nerve tissue but also aids in guiding axonal regeneration and connection to one of the neural ends to the other. In recent years, the 3d porous scaffold has been thought to be only providing cells with temporary support for cell growth [10]. However, modern scaffolds have evolved to be a critical factor in tissue engineering application, and these scaffolds have been demonstrated to be more than a mere container or support [11]. Therefore, they are also rich in growth factors, which would significantly enhance the regeneration of nerves [12]. Of which, Zhang conducted a 20-years study involving peripheral nerve defects, and the data collected were used to establish guidelines for studies involving peripheral nerve conduits [7]. The studies pointed out that neural lesions with a size of 9.7 ± 1.8 mm could successfully regenerate with the application of nerve conduits. However, for the repair of rat sciatic nerves that are >10 mm, the incorporation of several different proteins, such as laminin [13], fibronectin [14], and collagen [15], into the neural conduits are required for optimal nerve regeneration. 

In recent years, the rapid development and advancement of additive manufacturing technology have spurned off a wave of interest in related research and studies [16,17,18,19]. As compared to traditional structures and processes, such as foamed polyurethane, stencil sheet molding, solvent casting, or freeze-drying, 3D printing is a better solution to fabricate complex pore patterns and dimensions with high precision [8]. Digital light processing (DLP) is one of the technologies available for 3D printing [20]. DLP uses a photo-polymerization technique to cross-link photo-curable materials according to your computer-aided design (CAD). When the photo-curable bio-ink absorbs the ultra-violet (UV) ray, it generates active radicals or ionic groups, which in turn leads to the subsequent reaction of polymer polymerization and cross-linking [21]. Three-dimensional structure stereoscopic scaffold within a range of micrometers to nanometers can be prepared using DLP as it uses the light source as its curing energy [22]. 3D printing is the recent trend as it can be seen in recent years that various studies have utilized different 3D-printing techniques to fabricate bioscaffolds using various biomaterials, such as collagen, poly-caprolactone, and gelatin [23,24,25]. Many studies have attempted to apply 3D printing to fabricate and create alternatives to autologous implantations and biomimic model [26,27]. However, results from clinical trials and animal studies have not been promising enough to allow it to replace autologous implantations [28,29]. Therefore, novel approaches had since emerged to attempt to increase the feasibility of 3D printed conduits or scaffolds, and a recent novel approach was to use decellularized extracellular matrix (dECM) of nerves and implement them into the development of nerve bio-conduits for nerve regeneration [30,31,32]. The dECM was usually obtained by exposing harvested nerves with chemical or physical methods to remove unnecessary cellular components, thus only maintaining the structural proteins and several growth factors that were supposed to mimic the microenvironment of normal tissues [33,34]. With this concept in mind, current research had focused on developing a dECM-contained biomaterial that had both structural and biological behaviors, which were very similar to the native environment [35]. It was hypothesized that dECM retained critical structural components or motifs that are important to guide cellular differentiation and proliferation into the intended tissues. Thus, dECM can be added into the material without the need for any subsequent surface modification. However, the addition of dECM alone without any modification would reduce the printability of light-curable material and thereby limiting the chance of producing an ideal nerve conduit. Therefore, we chose polydopamine (PDA) because of its ideal oxidation reaction with the resin, thus allowing the composite to have an additional light absorbing capability. In recent years, PDA has been most commonly used for biomaterials modification due to its optimal cell adhesion behaviors [36]. In addition, PDA is also an excellent adherence promoter that can greatly improve the interface binding force of dECM and biomaterials, thereby preventing their segregation that might affect the regeneration of nerves [37]. 

This study aimed to fabricate water-based light-cured polyurethane (PU) nerve conduits with DLP technology. In order to improve the resolution and cytocompatibility of PU, homogenous polydopamine (PDA) and dECM was mixed into the raw material. PU belongs to a group of compounds termed as reaction polymers and is formed by exposing isocyanate and polyol to ultraviolet lights. The characteristics of PU can, therefore, be controlled by regulating quantities of isocyanate and polyol, making it an ideal material to be used for bioprinting. In addition, the novel idea of using PDA as a modification method was inspired by mussel’s attachment onto walls. The initial PDA-contained PU conduits functioned as a modified supportive material to allow efficient attachment and adhesion of dECM onto conduits. Mechanical properties and chemical composition were analyzed using the EZ Test machine (Shimadzu, Kyoto, Japan) and electron spectroscopy for chemical analysis (ESCA). In general, PU/PDA/dECM conduits were able to influence and enhance stem cell adhesion, proliferation, and neural differentiation.

## 2. Materials and Methods

### 2.1. ECM Extraction

ECM was harvested from rat sciatic nerves with appropriate and proven protocols [9]. The decellularization method used in this study had been described elsewhere but with slight modifications. Harvested nerves were minced into 1 mm thick pieces. The minced tissues were stirred in distilled water for 24 h with water changing twice daily. This step was critical in ensuring that the tissues were free of blood and debris. Step-up decellularization was done for the next 3 days. For the second day, tissues were stirred in 1% SDS with 1% Triton X-100 in PBS (1% PBS-T) for 24 h, and for the third day, tissues were stirred in 2% PBS-T containing 2% SDS for 24 h. For the fourth day, tissues were stirred in 3% PBS-T containing 3% SDS for 24 h. After this, the tissues were disinfected for 4 h with 0.1% peracetic acid in 4% ethanol. The tissues were then washed with distilled water for 1 h after disinfection. After this, the tissues were lyophilized and stored in the freezer for future use. 

### 2.2. Preparation of Water-Based PU 

Water-based PU resin (Alberdingk Boley, Krefeld, Germany) was stirred at 180 °C to allow dehydration. Specified amounts of 1.5% 2,4,6-trimethylbenzoyl-diphenyl-phosphineoxide (TPO, Ciba Specialty Chemicals Inc., Basel, Switzerland), 0.1% 2-hydroxy-4-methoxybenzophenone-5-sulfonic acid hydrate (HMBS, Tokyo Chemical Industry Co., Ltd. Tokyo, Japan), and 0.01% 4-hydroxy-2,2,6,6-tetramethyl-piperidinooxy (TEMPO, Sigma-Aldrich, St. Louis, MO, USA) were dissolved in 30% 2-Hydroxylethyl methacrylate (HEMA, Sigma-Aldrich) before being added to PU and stirred for 10 min to ensure homogeneity. In addition, 2 mg/mL dopamine (Acros Organics, Morris Plains, NJ, USA) was mixed with 1.2 mg/mL ammonium persulfate (Sigma-Aldrich, St. Louis, MO, USA) in pH 7 distilled water, added to the photocurable PU, and further dehydrated.

### 2.3. Preparation of PU/PDA/ECM Matrix 

The powdered dECM was enzymatically digested using 1 mg/mL of porcine pepsin in 0.01 N HCl solution. The mixture was then placed under a constant stir rate for 72 h. Neutralization was accomplished with the addition of 0.1 N NaOH. The dECM solution was added into the PDA/PU solution with a final concentration of 10 mg/mL.

### 2.4. Conduit Fabrication 

**Figure 1:**
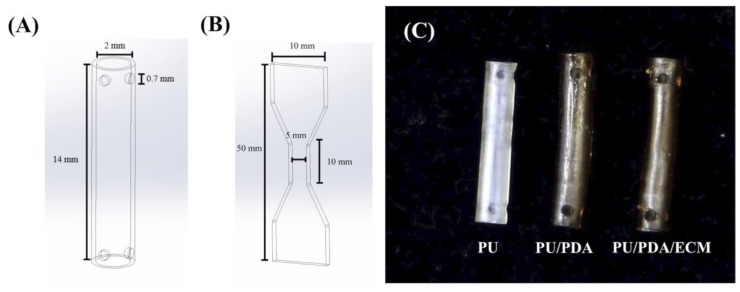
Schematic drawing of (**A**) the conduit and (**B**) the dumbbell-shaped sample used in the mechanical testing. (**C**) The top-view photograph of 3D-printed PU-based conduits. PU, polyurethane; PDA, polydopamine; ECM, extracellular matrix.

All 3d-printed conduits were drafted using SolidWorks (Dassault Systemes SolidWorks Corp., Waltham, MA, USA) and fabricated with MiiCraft high-resolution home DLP 3D printer (Young Optics Inc., Hsinchu, Taiwan). Each layer was designed to be at 100 μm per layer and exposed to 20 s of blue light digital stereolithography for curing. The characteristics of the water-based light-cured PDA/PU nerve conduits were set at 12 mm length, an outer diameter of 2.5 mm, and an inner diameter of 2 mm (Figure 1). The remaining materials were rinsed off by EtOH and exposed to UV light for post-curing to obtain the final conduits. After this, the conduits were rinsed and sterilized for in vivo implantations.

### 2.5. Physical Properties of 3D-Printed Conduits

The water contact angle test of each conduit was conducted at room temperature. In brief, the samples were situated on a platform, and 5 μL of MilliQ water was then pipetted onto the surface of each sample. A camera was used to capture images of the water droplet after 20 s. After this, ImageJ software from the National Institutes of Health was used to analyze and to measure the water contact angle. In addition, the mechanical stress-strain tests were done in dry environments and analyzed using a universal tensile tester. For this test, the samples were printed into a dumb-bell shape and subsequently stretched from both ends at a rate of 1 mm/min. Six assays were conducted for each sample, and the average was recorded. Fourier transform infrared spectroscopic analysis (FTIR) assay of the PU/PDA/ECM conduits was performed using an optical spectrometer in reflection absorption mode and within the wavenumber region of 600–2000 cm^−1^. In addition, 1 cm^−1^ of the spectral resolution was employed to measure the different functional groups on these conduits. Furthermore, electron spectroscopy was used to analyze the chemical compositions and elemental compositions of all specimens (ESCA, PHI 5000 VersaProbe, ULVAC-PHI, Kanagawa, Japan). The concentrations of the analyzed compositions were presented in the atomic percentage.

### 2.6. Biodegradation

To test for degradation, the conduits were immersed into simulated body fluid (SBF) at 37 °C for different durations. SBF used in this study was prepared with methods from our previously reported studies [25]. After soaking for various periods, the conduits were removed from SBF, washed, and dried for a day. Then, the dried conduits were weighed using the analytical balance (TE214S, Sartorius, Goettingen, Germany) and recorded to determine in vitro degradation behavior. Six tests were conducted for each time-point, with the average value tabulated. The SBF solution was similar to human blood plasma and consisted of 7.9949 g of NaCl, 0.2235 g of KCl, 0.147 g of K_2_HPO_4_, 0.3528 g of NaHCO_3_, 0.071 of g Na_2_SO_4_, 0.2775 g of CaCl_2_, and 0.305 g of MgCl_2_·6H_2_O in 1000 mL of distilled H_2_O with the pH adjusted to 7.4 using hydrochloric acid and trishydroxymethyl aminomethane.

### 2.7. Cytocompatibility of PU-Based Conduits Extracts

The indirect cytocompatibility was analyzed using a revised version of ISO10993-12. Briefly, the printed PU-based conduits were washed with PBS thrice and were disinfected in 75% ethanol for 1 h at room settings. After this, the different conduits were immersed in Dulbecco′s modified Eagle′s Medium (DMEM) and placed in a 37 °C incubator for 24 h with a pre-determined set of 75% humidity and 5% CO_2_. The DMEM was then used as extracts to test for cytocompatibility. Concurrently, cell culture of L929 cells, with a cell count of 10^4^ cells, was seeded in a 96-well at 37 °C for 24 h. Following this, the growth medium from the 96-well was removed and replaced with 200 µL of the various extracts. After different periods of cell culture, the extract solution was then removed and replaced with 100 µL of MTT solution (5 mg/mL) in each well. Following this, the culture was then left to be incubated for 3 h in the dark. Then, the MTT solution was removed and replaced with 100 µL of dimethyl sulphoxide for the dissolution of formed formazan, which was formed from the addition of MTT solution. A microplate reader (Infinite 200® PRO microplate reader, Tecan, Männedorf, Switzerland) was used to analyze the absorbances of each well.

### 2.8. Enzyme-Linked Immunosorbent Assay

Enzyme-linked immunosorbent assay (ELISA, Invitrogen, Grand Island, NY, USA) was used to analyze the concentrations of type I collagen (Col I) and laminin in the conduit extracts. ELISA was conducted in accordance with the manufacturer′s instructions. Subsequently, the levels of Col I and laminin in the conduit extracts were considered with correlation to the standards and standard curve. Six independent experimental analyses were performed for each specimen.

### 2.9. Cell Proliferation

All 3D-printed conduits were concurrently sterilized using 75% ethanol and ultraviolet light exposure for 30 min. The primary human Schwann cells (HSCs) used in this study were obtained from ScienCell Research Laboratories (Sciencell, San Diego, CA, USA) and cultured in Schwann cells medium (#1701, Sciencell) to cell passage 3–8. A density of 105 cells per specimens was directly seeded onto the specimens and placed in a 37 °C, 5% CO2 incubator for various durations. After 1, 3, and 7 days of culture, Prestoblue® (Invitrogen, Grand Island, NY, USA) reagent was used to evaluate and analyze cell quantities. Briefly, the culture medium was removed, and the specimens were rinsed several times with cold PBS. Following this, a ratio of 1:9 PrestoBlue® and a fresh medium was used to fill each specimen. Subsequently, the reaction solution was incubated for 90 min at 37 °C before the analysis of PrestoBlue® absorbance. In brief, the reaction solutions were removed from the specimen and placed on a new 96-well plate. Absorbance was measured by using a Tecan Infinite 200® PRO microplate reader at 570 nm with a reference wavelength of 600 nm. Data for this study were done in triplicates from three different cultures, and HSCs cultured on culture plates without any specimens were used as a control (Ctl). In addition, F-actin staining was conducted for cell morphology observations. After 7 days of culture, the conduits were rinsed thrice with PBS before being fixated with 4% paraformaldehyde for 20 min. Following this, the conduits were placed into 0.1% Triton X-100 at room temperature for 15 min to permeate the cells. Then, the specimens were incubated with AlexaFluor-488-conjugated phalloidin for 1 h, and DAPI (4′,6-diamidino-2-phenylindole, dilactate) was used to stain for the nucleus. For DAPI, the fixated cells were placed in DAPI for 20 min in the dark at room temperature. The images were then photographed using Leica TCS SP8 X white light laser confocal microscope (Leica Microsystems GmbH, Wetzlar, Germany). 

### 2.10. Cell Differentiation

For neurogenic-related protein assays, the seeded HSCs were lysed with NP40 buffer (ThermoFisher, Waltham, MA, USA) after 7 days of cell culture. A BCA protein assay kit was used to analyze total protein concentrations. Briefly, cell lysates (40 μg protein/sample) were segregated using sodium dodecyl sulfate-polyacrylamide-polyacrylamide gel electrophoresis before being transferred over to PVDF membranes (Millipore, Billerica, MA, USA). A 2% bovine serum albumin (Invitrogen, Carlsbad, CA, USA) in tris-buffered saline with 0.1% Tween 20 was used to fixate the proteins on the membranes before immunoblotting with primary Nestin, MAP2 (microtubule-associated protein 2), and β-actin (Abcam, Cambridge, MA, USA) antibodies for 2 h. Following the immunoblotting, the membranes were incubated with horseradish peroxidase (HRP)-conjugated secondary antibodies to allow chemiluminescence and physical visualization of bands using an ECL detection kit (Invitrogen). For each protein, their expression levels were normalized to β-actin. Finally, we also used ELISA to determine the concentration of Nestin and MAP2 protein production.

### 2.11. Immunofluorescence Staining

The HSCs were grown on different conduits for 7 days. Then, the conduits with cells were rinsed using PBS and subsequently fixed by placing them in 4% paraformaldehyde (Sigma-Aldrich) for 20 min at normal room temperature. After this, the cells were permeabilized using 0.1% PBS-T, immune-stained with anti-β-tubulin III (Abcam), and then with anti-mouse conjugated tetramethylrhodamine (TRITC, Invitrogen). The phalloidin affixed with Alexa Fluor 488 (1:300 dilution in PBS, Invitrogen) was used to stain the intracellular F-actin filaments. The immunofluorescence staining results of the HSCs on the conduits were observed under a Leica TCS SP8 X white light laser confocal microscope (Leica Microsystems GmbH, Wetzlar, Hessen, Germany).

### 2.12. Statistical Analyses

All data are presented as mean ± standard deviation (SD) and were compared by a one-way analysis with Scheffe′s multiple comparison post hoc test. In all cases, the results were considered to be statistically significant with a *p*-value <0.05.

## 3. Results and Discussion

### 3.1. Characterizations of PU/PDA/ECM Conduits

**Figure 2:**
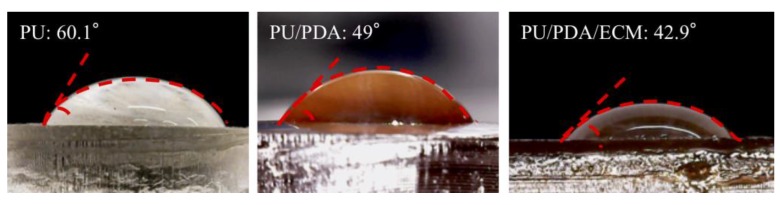
The water contact angle of PU, PU/PDA, and PU/PDA/ECM specimens.

The hydrophilicity of a conduit has a critical role to play in influencing cellular adhesion and proliferation, which would further enhance downstream cellular behaviors. Reports had been made stating the positive effects of hydrophilicity on cellular activities. Hydrophilicity can be determined by measuring the static water contact angle between the water droplet and the surface of the different conduits, as shown in Figure 2. PU had the highest water contact angle of 60.1 ± 2.9° as compared to PU/PDA (49.0 ± 1.5°) and PU/PDA/ECM conduit (42.9 ± 1.8°). Due to the addition of PDA to the PU matrix, the contact angle was reduced by 18% in comparison with the PU material (*p* < 0.05). Also, the incorporation of dECM reduced the contact angle by 12% compared to PU/PDA (*p* < 0.05), and thus, the contact angle of PU/PDA/dECM group was in the range of contact angles for maximal cell adhesion [38]. These results indicated that the addition of PDA and ECM was able to enhance hydrophilicity as demarcated with a lower contact angle. It was important to note that hydrophilicity was enhanced with PDA alone and further improved with the addition of ECM. This result was consistent with our previous studies and also in agreement with various reports, indicating that the addition of PDA and ECM enhanced hydrophilicity [39]. It was hypothesized that the introduction of ECM provided many hydrophilic amines and hydroxyl groups onto the surface, thus increasing the hydrophilicity of the conduit. In general, the hydrophilicity of the conduits was increased with the addition of ECM, and enhanced hydrophilicity was reported to promote cellular adhesion and other cellular behaviors [40].

**Figure 3:**
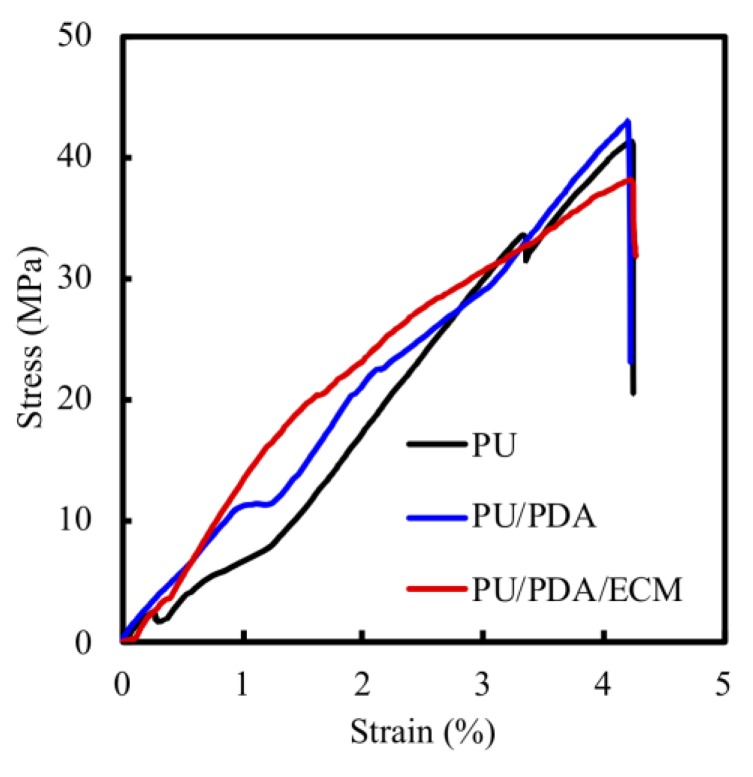
The tensile stress-strain curve of 3D-printed PU-based specimens.

Figure 3 shows the tensile stress-strain curves of the various conduits. The strain rates were fixed for all three conduits. Upon reaching their maximal stress-strains, all the conduits were torn in the middle of the conduits without much differences in tearing locations. The maximal tensile strength achieved for each of the conduits were as follows: PU (42.0 ± 2.1 MPa), PU/PDA (43.1 ± 1.8 MPa), PU/PDA/ECM (38.8 ± 1.6 MPa), and there were no significant differences between these three nerve conduits. From the results collected, PU/PDA conduit exhibited the highest maximal tensile strength, followed by the PU conduit, while the PU/PDA/ECM conduit exhibited the lowest maximal tensile strength with approximately 10% lower tensile strength as compared to the rest. However, even though PU/PDA/ECM displayed the lowest maximal tensile strength amongst all the conduits, this difference was insignificant as it still meets the optimal requirement of a nerve conduit for proper surgical handlings and implantations. In previous studies, it was demonstrated that the elastic modulus of the human nerve was similar to the stress-strain behavior of rat nerves (13.8 ± 5.4 MPa) [41,42]. Therefore, the above data indicated that the PU/PDA/ECM conduit had sufficient mechanical strength to undergo surgical manipulations and implantations.

**Figure 4:**
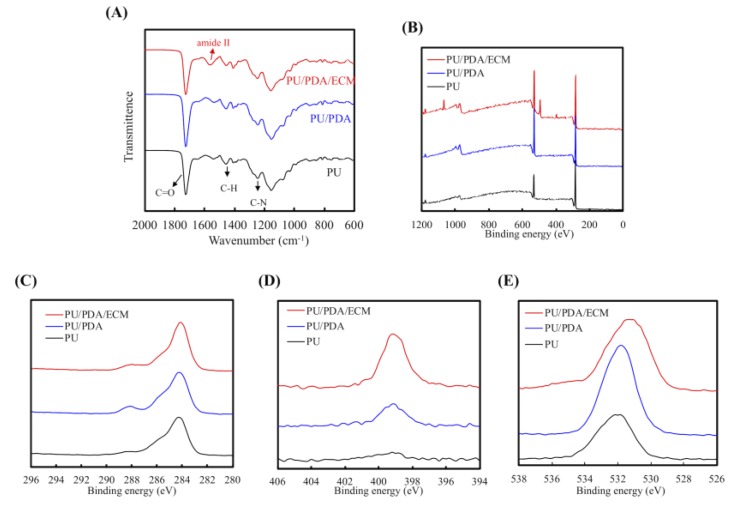
(**A**) FTIR spectra, (**B**) XPS, (**C**) C1s, (**D**) N1s, and (**E**) O1s high-resolution spectra of PU, PU/PDA, and PU/PDA/ECM composites.

In Figure 4A, all specimens show the presence of absorption peaks assigned to C–H bending vibration (1453 cm^−1^) and C–N stretching vibrations (1244 cm^−1^). In addition, PU-based materials also displayed one strong peak at 1732 cm^−1^ that was associated with non-H-bonded urethane carbonyls [43]. In addition, PU/PDA/ECM presented protein-related functional groups (amide II bands) at 1595 cm^−1^ [44]. Figure 4 displays the XPS spectra obtained from different conduits, and the results showed that PDA and dECM could be successfully fused with PU. From Figure 4B, it can be seen that there was an increment in photoelectron peaks of C1s (Figure 4C), N1s (Figure 4D), and O1s (Figure 4E), thus further indicating the successful incorporation of PDA and dECM to the PU. The successful fusion of PDA onto PU was indicated with the higher nitrogen peaks, and successful fusion of maleimide and dECM was indicated by the increase of N–C=O component at a binding energy of 289 eV. The XPS spectra showed little differences in their overall structure of binding energy waves, thus indicating that the original compositions of PU were preserved despite the additions of PDA and dECM (Figure 4A). The increase of the N–C=O component at 289 eV indicated the introduction of maleimide and ECM bonds on the surface [45]. This was important to note as it indicated that the printability of PU remained unaffected despite the addition of PDA and dECM. Overall, these data showed that PDA and dECM were able to be successfully implemented onto PU and most critically, not affecting the original structural compositions of PU.

**Figure 5:**
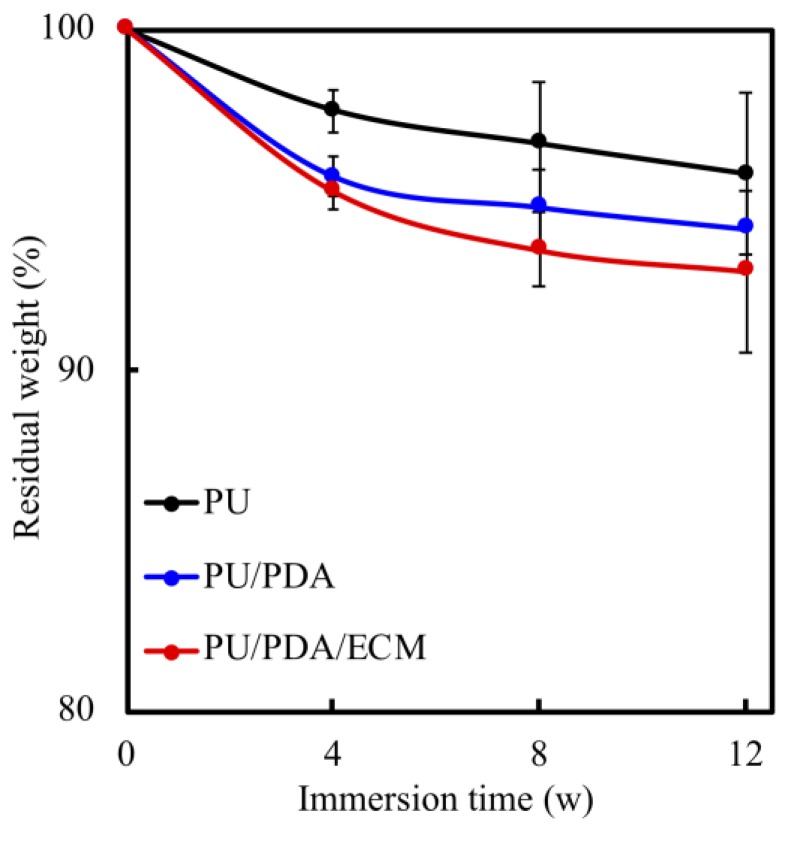
The degradation rate of various 3d-printed PU-based conduits after immersion in SBF (simulated body fluid) for different time-points.

The degradability of material must be taken into consideration when developing nerve conduits as it plays a critical role in biodegradation, and thus affecting the overall efficacy. A suitable nerve conduit should have a biodegradation rate that matches the rate of neural regeneration that allows proper and efficient nerve repair. Thus, the degradation rates of each conduit immersed in SBF were recorded from different time points, as shown in Figure 5. All specimens showed increased degradation with increased immersion time. The steepest degradation rates for all specimens were seen during the first 4 weeks of immersion with a maximal weight loss of 2–4% depending on the different types of conduits. The degradation rates of all specimens slowed down after week 4 and had gradual degradation rates until week 12. At the end of the immersion tests (week 12), the PU, PU/PDA, and PU/PDA/ECM conduits had weight losses of 4.23%, 5.86%, and 7.08%, respectively. As seen from this study, PU/PDA/ECM conduit had the highest degradation, followed by PU/PDA and PU conduits. According to studies, the time taken for nerve regeneration is approximately 6 to 12 weeks depending on the severity; therefore, logically, a nerve conduit should be able to last at least 12 weeks to allow complete regeneration. A detailed report on the MRI results of nerve degeneration and regeneration mentioned that nerve functions were fully restored 12 weeks after the onset of nerve damage [46]. Our data showed that our conduits had 93%–96% of residual weights by the end of week 12, thus demonstrating that three conduits maintained structure until the complete regeneration of nerve tissue.

### 3.2. Cytotoxicity of PU/PDA/ECM Conduits

**Figure 6:**
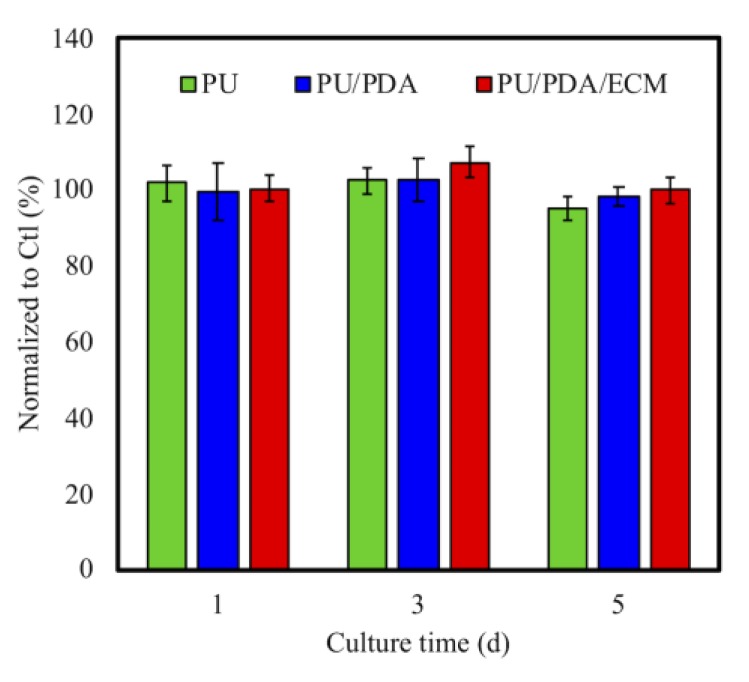
Quantification of a cytotoxic test of extract solutions of PU, PU/PDA, and PU/PDA/ECM conduits relative to controls on L929 cells for different time-points.

Direct and indirect cytotoxic tests exist to test for cytotoxicity of biomaterials. Indirect testing was used in this study, and it was especially a critical method to assess the toxicity of degrading biomaterials and its released byproducts. ISO 10993-12 cytotoxicity test was used in this study. The results were presented as the percentage of viable L929 cells in the PU, PU/PDA, and PU/PDA/ECM conduits extracts, which represent a pure cell culture of L929 cells (Figure 6). Cells cultured on culture dish with the normal medium were used as controls. No significant cytotoxicity was noticed amongst all three groups. It should be noted that the 3D-printed PU-based conduits can provide a favorable microenvironment for cell cytocompatibility [47]. Furthermore, it was hypothesized that the addition of PDA and dECM, which were both natural compounds, led to decreased immunogenicity of PU, thus making it a more suitable candidate for in vivo implantations [48]. 

### 3.3. The Nerve-Regeneration-Promoting Growth Factor of Different Conduits

**Figure 7:**
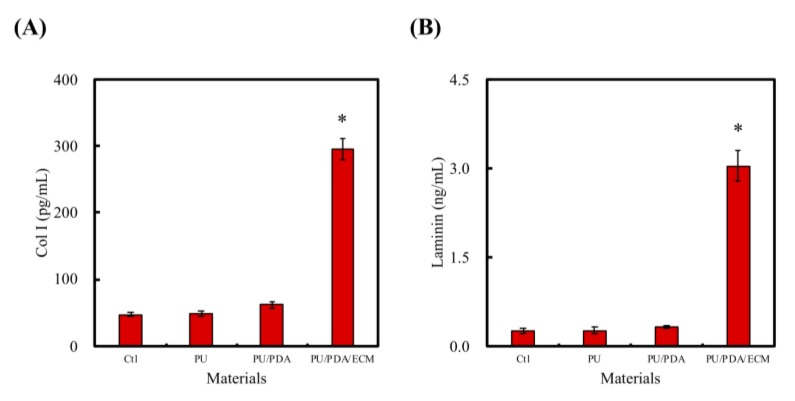
(**A**) Col I (type I collagen) and (**B**) laminin were released from PU, PU/PDA, and PU/PDA/ECM conduits in DMEM extracts. * indicates a significant difference (*p* < 0.05) compared to Ctl (control).

Laminin is a component of ECM that can be found in the nervous system and is critical in neural development, such as neuronal migration, myelination, the formation of the neuromuscular junction, as well as peripheral nerve regenerations. The other critical component, collagen, which functions as anchoring points for various proteins and enzymes, also has an important role to play in nerve regenerations. Laminin and Col I were quantified using ELISA protein assays, as shown in Figure 7. As seen, the amount of both Col I and laminin were significantly higher in PU/PDA/ECM conduit as compared to PU and PU/PDA conduits. The Col I amount from each of the groups (Ctl, PU, PU/PDA, PU/PDA/ECM) were as follows: 46.9 ± 2.9 pg/mL, 49.4 ± 3.9 pg/mL, 61.8 ± 3.5 pg/mL, 295.2 ± 15.3 pg/mL, respectively. On the other hand, the amounts of laminin from each of the groups (Ctl, PU, PU/PDA, PU/PDA/ECM) were as follows: 0.25 ± 0.05 ng/mL, 0.28 ± 0.05 ng/mL, 0.32 ± 0.03 ng/mL, 3.04 ± 0.26 ng/mL, respectively. It was worthy to note that PU/PDA/ECM had approximately 11-times higher laminin and 6-times higher Col I as compared to PU. PDA had been shown, and proven by others, to act as an efficient adherence promoter, allowing enhanced dECM adherence onto PU conduits and other biomaterials [49]. Of which, laminins are the most significant class of ECM proteins in the nervous systems, and they have been reported to have critical roles in supporting various functions, such as neuronal migration, myelination, and formation of neuromuscular junctions [50]. On the other hand, Col is the most abundant and critical ECM protein that mainly functions as structural anchoring points for various enzymes or proteins. These results showed that PU/PDA/ECM nerve conduits could release factors that would promote nerve regeneration, and subsequent evaluation would be conducted to further determine the feasibility of this material in the differentiation and regeneration of the nerve cells.

### 3.4. Cell Proliferation and Morphology 

**Figure 8:**
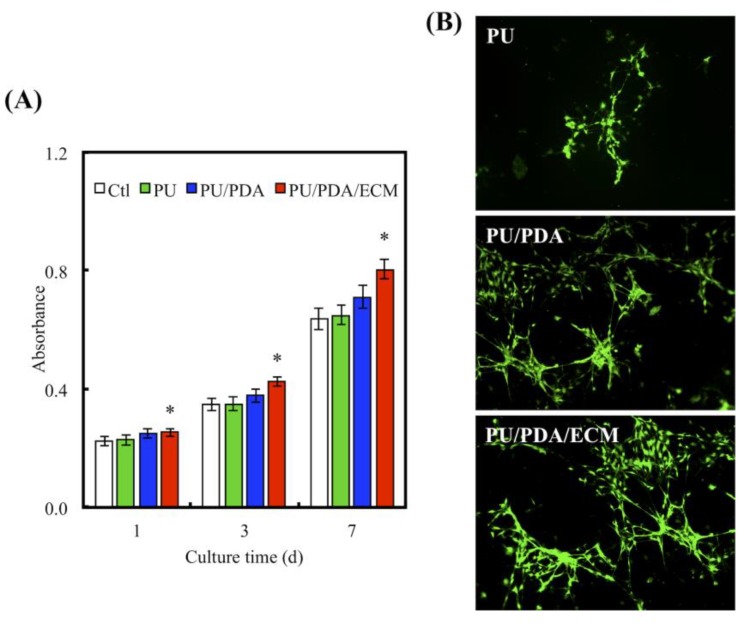
(**A**) Proliferation of HSCs (human Schwann cells) cultured on PU, PU/PDA, and PU/PDA/ECM conduits for 1, 3, and 7 days. * indicates a significant difference (*p* < 0.05) compared to Ctl. (**B**) F-actin staining of HSCs seeding on PU, PU/PDA, and PU/PDA/ECM conduits for 3 days.

Figure 8A shows the quantification of the primary human Schwann cells (HSCs) proliferation after 1, 3, and 7 days of culture on the various conduits. PU displayed approximately similar proliferation rates as compared to Ctl, while PU/PDA was shown to have higher proliferation rates as compared to both Ctl and PU groups. In this case, only the PU/PDA/ECM group exhibited significantly higher cellular proliferation rates at all time-points as compared to the rest of the groups (*p* < 0.05). PU/PDA/ECM conduits had approximately 20%, 19%, and 15% higher proliferation as compared to Ctl, PU, and PU/PDA groups, respectively. The morphology of the adhered cells was also shown in Figure 8B. It can be observed that cells cultured on the PU conduits exhibited a more clustered and shrouded appearance as compared to the other groups. There were not many cellular protrusions and spreading, thus indicating that the cells were not properly adhered to the surfaces of the conduits. On the other hand, HSCs on PU/PDA/ECM showed evidence of cytoplasmic spreading via its processes. Cells were flattened and well spread as compared to the other groups. In addition, areas covered with cells were evidently increased, thus indicating increased proliferation, which was in good agreement with the quantified results from Figure 8B. It was hypothesized that enhanced hydrophilicity caused by the addition of ECM could contribute to the enhanced adhesion and proliferation, as studies have shown that hydrophilicity enhances cellular adhesion, thus leading to increased cellular proliferation [40]. Furthermore, the addition of ECM, a major component of native tissue, could also up-regulate adhesion and proliferation by providing native moieties for cells to adhere to. Interestingly, these results were consistent with the observations made by others, whereby the combination of natural proteins or polysaccharides with synthetic polymers were able to enhance cell adhesion [51]. In fact, HSCs had a rapid response to axonal injury, with an estimated time of response prior to the beginning of axonal degeneration. They recruit macrophages by releasing chemokines and cytokines to aid in improving the rate of myelin clearance. Our results support that the 3d-printed PU/PDA/ECM nerve conduits act as a bridge and shorten the gaps between two blunt nerve fiber endings [8].

### 3.5. Neurogenic-Related Protein Expression 

**Figure 9:**
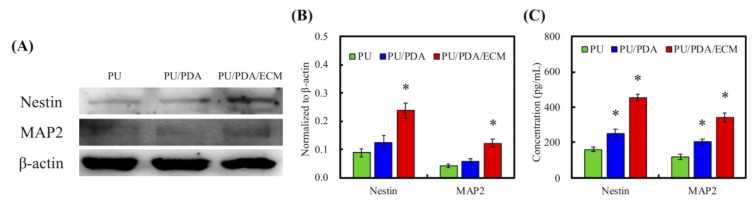
(**A**) The western blot pattern of neurogenic-related protein, Nestin, and MAP2 in HSCs cultured with 3D-printed nerve conduits for 7 days. The quantification of neurogenic-related protein from (**B**) western blot and (**C**) ELISA, respectively. Data presented as mean ± SEM, n = 3 for each group. “*” indicates a significant difference (*p* < 0.05) when compared to PU conduit.

**Figure 10:**
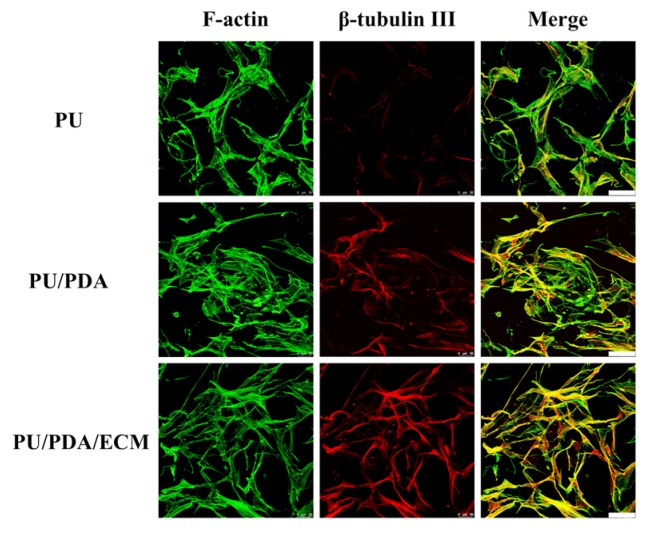
The immunofluorescence staining of β-tubulin III (**red**) and F-actin (**green**) of HSC cells grown on PU, PU/PDA, and PU/PDA/ECM conduits after cultured for 7 days.

The secretion levels of Nestin, MAP2, and actin were examined using western blot to further assess the status of cellular adhesion (Figure 9A). Nestin is a type of intermediate filament protein and is highly expressed during neural development that could be considered as an HSC specific marker [52]. From Figure 9B, it could be seen that PU/PDA/ECM induced higher expression of Nestin and MAP2 as compared to PU and PU/PDA (*p* < 0.05). The result from ELISA in Figure 9C was also similar to those in Figure 9B. The ECM-containing nerve conduit was more effective in promoting the secretion of neural differentiation proteins. The results of ELISA showed that the performance of Nestin and MAP2 in PU/PDA/ECM was 2.8 and 2.9-times higher than that of PU, respectively. Moreover, the immunofluorescence staining results with β-tubulin III nerve proteins (Figure 10) also demonstrated that the 3D-printed PU-based conduits supported neural differentiation, with the neurite extension higher in PU/PDA/ECM conduits than PU conduits. Its expression is usually transient and is only expressed during tissue development or during pathological injuries to promote regeneration. On the other hand, MAP2 is a protein that aids in microtubule assembly, which is critical in neurogenesis as it allows the formation of new neurites, which would eventually develop to become axons and dendrites. The secretion levels of Nestin and MAP2 of HSCs were similar between PU and PU/PDA conduits. In contrast, HSCs cultured on PU/PDA/ECM conduits had significantly higher secretions of Nestin and MAP2 as compared to the other groups, indicating that PU/PDA/ECM conduits were able to enhance neural development and regeneration. Both Nestin and MAP2 are commonly classified as neuronal differentiation markers whereby their level of expression would be greatly down-regulated after neuronal differentiation and maturation [53]. On the other hand, MAP2 is a protein found only in the cytoskeleton of mature neural cell, and thus HSCs usually has a low expression of MAP2 during early stages of differentiation. A gradual increment of MAP2 can be seen as neuronal cell mature and progress along the neuronal development process.

## 4. Conclusions

In summary, we successfully fabricated a PU/PDA/ECM nerve conduit in this study. Our initial studies had attempted to print PU/ECM directly, but we failed to produce nerve conduits using this way. However, for the subsequent trial, the printability of PU-resin was significantly improved with the addition of PDA into PU. In addition, it was critical to note that the addition of PDA/ECM did not inhibit the original mechanical properties of PU, thus allowing us to retain the advantages of PU. PU/PDA/ECM conduits and their degraded byproducts were found to be non-cytotoxic to L1929 cells, thus making it a suitable candidate for implantations. It was also found that even though PU/PDA/ECM conduits had slightly lower mechanical properties as compared to their counterparts, the difference was insignificant as the mechanical properties were still well within the range of surgical manipulations and implantations. Furthermore, PU/PDA/ECM conduits were reported to be more hydrophilic as compared to PU or PU/PDA conduits, thus allowing enhanced cellular adhesion as seen from the F-actin stains. Numerous studies have reported that the quality of cellular adhesion is directly proportionate to downstream cellular behavior and activities, such as adhesion, proliferation, and differentiation. Similarly, we were able to see the significant increment in HSC proliferation on PU/PDA/ECM conduits as compared to the rest of the groups. Furthermore, PU/PDA/ECM conduits were able to significantly increase the secretions of neural-related proteins (Col I and laminin) and also enhance the expression of neurogenic proteins (Nestin and MAP2). Therefore, we hypothesized that PU/PDA/ECM conduits were able to efficiently support neural regeneration, thus making them a potential applicant in future nerve-related studies or clinical applications.
